# Microbiology of secondary infections in Buruli ulcer lesions; implications for therapeutic interventions

**DOI:** 10.1186/s12866-020-02070-5

**Published:** 2021-01-05

**Authors:** Elizabeth Gyamfi, Charles A Narh, Charles Quaye, Adiza Abbass, Bartholomew Dzudzor, Lydia Mosi

**Affiliations:** 1 Department of Medical Biochemistry, University of Ghana Medical School, Korle Bu, Accra, Ghana; 2 Department of Biochemistry, Cell and Molecular Biology, University of Ghana, Legon, Accra, Ghana; 3 West African Center for Cell Biology of Infectious Pathogens, University of Ghana, Legon, Accra, Ghana; 4 Noguchi Memorial Institute for Medical Research, University of Ghana, Legon, Accra, Ghana; 5 Burnet Institute for Medical Research, Melbourne, Australia

**Keywords:** *Mycobacterium ulcerans*, Buruli ulcer, Secondary Infection, Antimicrobial resistance, Antibiotics

## Abstract

**Background:**

Buruli ulcer (BU) is a skin disease caused by *Mycobacterium ulcerans* and is the second most common mycobacterial disease after tuberculosis in Ghana and Côte d’Ivoire. *M. ulcerans* produces mycolactone, an immunosuppressant macrolide toxin, responsible for the characteristic painless nature of the infection. Secondary infection of ulcers before, during and after treatment has been associated with delayed wound healing and resistance to streptomycin and rifampicin. However, not much is known of the bacteria causing these infections as well as antimicrobial drugs for treating the secondary microorganism. This study sought to identify secondary microbial infections in BU lesions and to determine their levels of antibiotic resistance due to the prolonged antibiotic therapy required for Buruli ulcer.

**Results:**

Swabs from fifty-one suspected BU cases were sampled in the Amansie Central District from St. Peters Hospital (Jacobu) and through an active case surveillance. Forty of the samples were *M. ulcerans* (BU) positive. Secondary bacteria were identified in all sampled lesions (*N* = 51). The predominant bacteria identified in both BU and Non-BU groups were *Staphylococci* spp and *Bacilli* spp. The most diverse secondary bacteria were detected among BU patients who were not yet on antibiotic treatment. Fungal species identified were *Candida* spp, *Penicillium* spp and *Trichodema* spp. Selected secondary bacteria isolates were all susceptible to clarithromycin and amikacin among both BU and Non-BU patients. Majority, however, had high resistance to streptomycin.

**Conclusions:**

Microorganisms other than *M. ulcerans* colonize and proliferate on BU lesions. Secondary microorganisms of BU wounds were mainly *Staphylococcus* spp, *Bacillus* spp and *Pseudomonas* spp. These secondary microorganisms were less predominant in BU patients under treatment compared to those without treatment. The delay in healing that are experienced by some BU patients could be as a result of these bacteria and fungi colonizing and proliferating in BU lesions. Clarithromycin and amikacin are likely suitable drugs for clearance of secondary infection of Buruli ulcer.

## Background

Buruli ulcer (BU) is a necrotizing skin disease caused by *Mycobacterium ulcerans* (MU). It is characterized by a painless nodule, papule, plaque or edema, which can develop into a painless ulcer with undermined edges, often leading to overturning sequelae and in rare cases, osteomyelitis [1]. The exact mode of transmission of the disease remains unclear. Globally, it is the third most common disease caused by mycobacteria after tuberculosis and leprosy [2]. Buruli ulcer remains a public health problem in Ghana; particularly, in the Amansie West and Central Districts [3].

The pathology of MU is different from other mycobacterial pathogens; it is primarily extracellular and harbors a plasmid which encodes mycolactone; a polyketide-derived macrolide toxin [4]. Cellular activities of mycolactone accounts for majority of the pathogenesis related to the disease. It also suppresses the host’s immune features from eliciting inflammatory responses [5], which explains why the majority of BU patients feel no pain. Pain experienced by BU patients have been associated with wound dressing and physiotherapy [6, 7] and underlying secondary infection [8] which is enhanced by the toxins ability to disrupt protective barriers of the skin [9]. Both pathogenic and opportunistic microbes, stemming from normal skin flora or the immediate environment, could colonize skin ulcers and cause secondary microbial infections among BU patients [10, 11].

Even though MU can infect any part of the body, lesions predominantly localize on the extremities (especially the lower limbs) due to the comparatively cooler temperature which favors the limited optimum growth of MU at 32^o^C. This may also be the result of these parts frequently being in contact with microbial contaminated environments and thereby, increasing the risk of bacterial infection of BU wounds [12]. The cytotoxic effect of mycolactone including destruction of skin and subcutaneous fat may provide fertile grounds for the colonization and proliferation of microorganisms from normal skin flora and pathogenic species from the environment which may result delayed in wound healing.

Even though mycolactone was initially thought to inhibit the growth of other bacteria on BU lesions, recent reports have identified secondary bacterial infections of BU wounds in both pre-treatment, during treatment, and post treatment [10, 13, 14]. Few studies have highlighted fungal element associated with BU [15]. There is, albeit insufficient information on the effect of antibiotics on secondary microorganism present in BU wounds. Microbial infections generally, have insignificant influence on rapidly healing wounds but may create large colonies on slow healing wounds [16]. Many wound and skin infections that complicate skin lesions are caused by mixed bacterial flora where they survive synergistically [17]. Interestingly, wound colonization by yeasts and fungi occasionally occur after treatment has usually begun [18]. There is also a paucity of information on the effect of the recommended antibiotics for BU treatment on secondary microorganism present in BU lesions [19].

BU has been recognized as a health problem in Ghana and various researchers have investigated ways in which antibiotic treatment and wound management can be improved. Understanding the diversity and role of microbial secondary infections in BU lesions is crucial to improving treatment efficacy. This study focuses on microbiological and molecular based identification of secondary microbial organisms from suspected BU patients and their antimicrobial resistant phenotypes.

## Results

### Demographic information on study cohorts

**Fig. 1:**
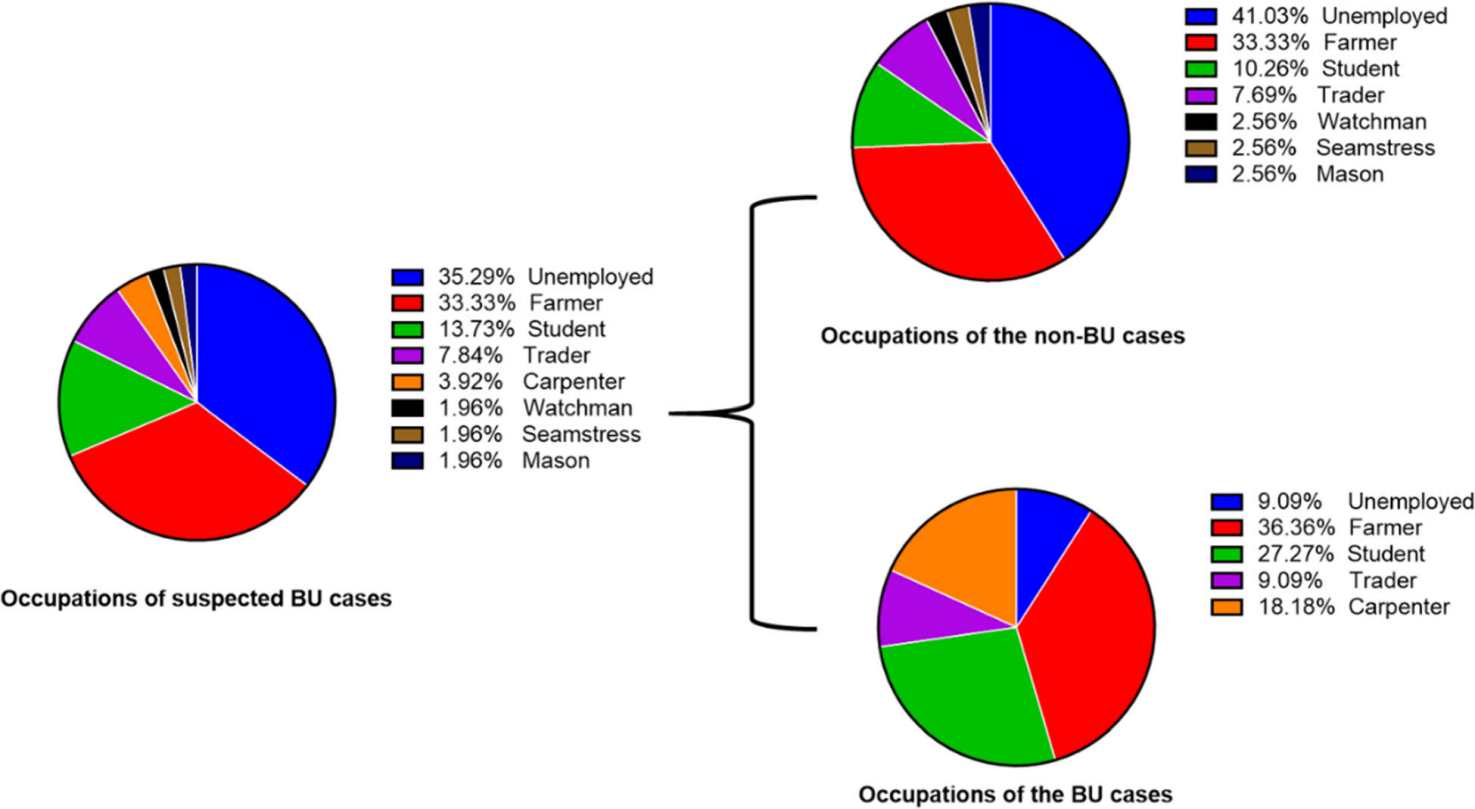
Occupations of participants recruited for this study. Farming was identified as the main occupation of all suspected BU cases in this study

Out of the 51 study participants 50.98% and 49.02% were females and males, respectively. Majority of the participant were adults with a total of 11 (21.6%) being below the age of 18 years; 20 (39.2%) participants were between 18 and 50 years and 20 (39.2%) above 50 years. The youngest and oldest persons were males; with the youngest being a year old and the oldest being 80 years. Over 35% of respondents practiced farming and other agricultural activities as their main occupation (Fig. 1).

### Buruli ulcer case confirmation

**Fig. 2:**
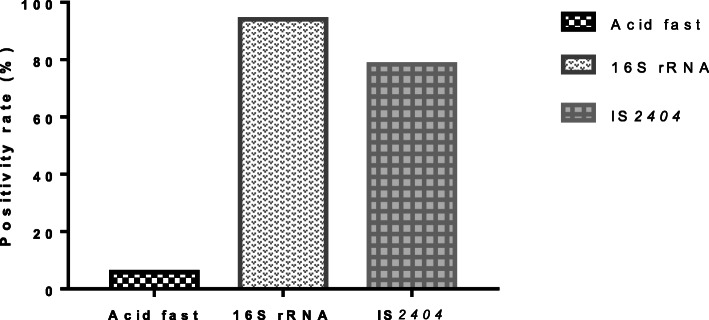
Acid-fast and PCR positivity among the suspected BU cases. All patient lesion samples were screened for the presence of acid-fast bacteria, mycobacterial 16SrRNA and IS*2404* gene amplification for BU case confirmation. A higher positivity rate was observed for PCR (16SrRNA & IS*2404*) compared to direct smear microscopy (detection of acid-fast bacilli) for the confirmation of BU cases

To confirm the presence of *Mycobacterium ulcerans* and hence, BU positive cases, all 51 samples were first screened for acid-fast bacilli using the acid-fast staining technique and amplification of the 16SrRNA gene specific for *Mycobacteria spp*. The samples positive for Mycobacteria were further screen for *M. ulcerans* by the amplification of IS*2404*. Microscopic detection of acid-fast rod-shaped bacteria was generally low and only 0.06% (3/51) of the samples were positive. Detection of Mycobacteria by amplification of the 16S rRNA gene was high with 94.1% (48/51) of the samples testing positive. Based on the amplification of IS*2404*, 40 samples (78.4%) were confirmed as *M. ulcerans* (BU) positive and 11 (21.6%) were negative for *M. ulcerans* DNA (non-BU) (Fig. 2).

**Fig. 3:**
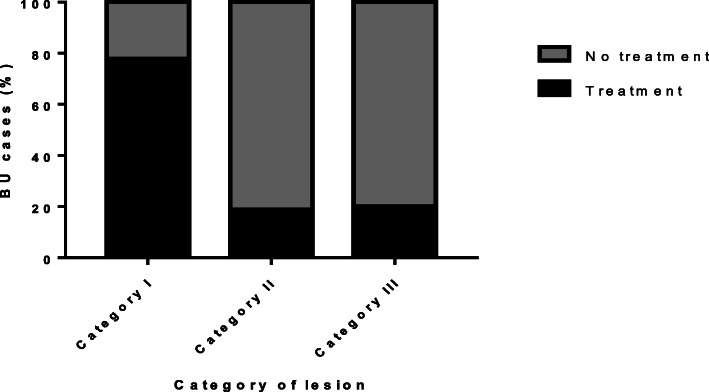
Category of BU lesions with or without treatment. Rifampicin and streptomycin were used at the health centres for BU treatment. A higher percentage of untreated BU patients were in category II and category III compared with category I

Out of the 40 positive cases, 14 (35%) had already commenced BU antibiotic treatment (BT) with rifampicin and streptomycin and wound dressing before we began this study and 26 (65%) had not (BNT). A total of 12.2% (5/40) of the BU participants on treatment had undergone surgery associated with Buruli ulcer. Based on lesion category, as directed by WHO BU guidelines[20], 22.5% (9/40) were category I (> 5 cm in width), 40% (16/40) were category II (≤ 5 cm-15 cm) whilst 37.5% (15/40) were category III (> 15 cm) lesion (Fig. 3).

A higher percentage of BU patients under treatment were in category I compared to category II and III. A significant difference was observed between treatment of BU and category of lesions (chi-square test of association; df 2, *p* = 0.004). Therefore, BU patients who sought the recommended treatment for Buruli ulcer at an early stage were less likely to migrate from category I (Fig. 3).

### Characteristics of isolated secondary microorganisms from BU wounds

**Fig. 4:**
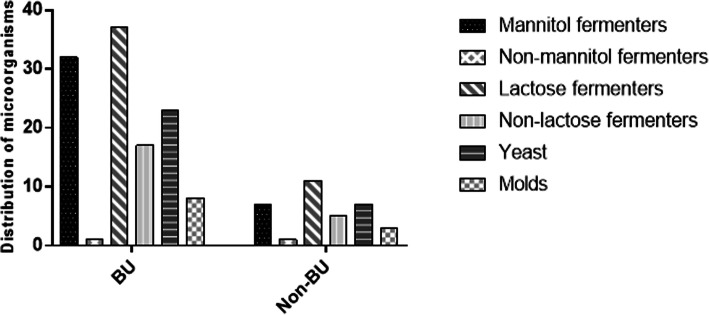
Secondary microbes identified through culture dependent methods. Data is representative of the total number of microbial isolates recovered from each participant’s (both BU positive lesions and Non-BU lesions) sample after culturing on Sabouraud dextrose agar, Mannitol agar and Mac Conkey

Plate cultures were performed for all 51 samples to isolate microorganisms present in the lesions. All the LB agar plates (*N* = 51: 40 BU and 11 non-BU samples) had bacterial growth. Of the 40 BU positive samples cultured on mannitol salt agar, growth was observed on 33 (83%) of the plates, indicating the presence of either *Staphylococci* or *Micrococci* spp in the lesions. Out of these, 32/33 (97%) were mannitol fermenters (pathogenic species) whilst 1/33 (3%) was a non-mannitol fermenter (non-pathogenic species). Mannitol fermenters were observed on 22/26 (84.6%) of BNT group and 11/14 (78.6%) of the BT group. For the BU negative samples, mannitol fermenters (pathogenic species) were observed on 7/11 (63.6%) whiles non-mannitol fermenters were observed on 1/11 (9%). A mixed infection (both mannitol and non-mannitol fermenters) was observed on 1 plate (Fig. 4).

Of the forty (40) BU positive samples cultured on MacConkey agar, growth was observed on 37 (93%) of the plates, indicating the presence of either gram negative or enteric bacteria. All the 37/40 (93%) had growth of lactose fermenters while 17/37 (43%) had growth of non-lactose fermenters. These results indicate the presence of mixed and/or multiple infections (both lactose and non-lactose fermenters) on some of the wounds (45.9%%). All the BNT samples 26/26 (100%) had the growth of lactose fermenters whiles BT samples had 11/24 (78.6%). For the non-lactone fermenters observed, growth was observed in 14/26 (53.8%) of the BNT samples and 3/14 (21.4%) of the BT samples. These results indicate the presence of mixed and/or multiple infections (both lactose and non-lactose fermenters) in some of the lesions (45.9%%). For the BU negative samples, lactose fermenters were observed on all 11 samples (100%). Non-lactose fermenters were observed on 5/11 (45.4%) plates, indicating the presence of mixed infection (both lactose and non-lactose fermenters) in 5/11 (45.4%) of the samples (Fig. 4).

To detect fungal species, the samples (*N* = 40 BU-positive samples) were cultured on Sabouraud dextrose agar. Growth of fungal elements was observed on 25/40 (62.5%) plates. Out of these, 23/35 (92%) were identified as yeast whiles 8/25 (32%%) were mold. Mixed infections of both yeast and mold were observed in 6/25 (24%) of the agar plates. Of the total number of yeasts observed, 13/26 (50%) were samples from the BNT group whiles 10/14 (71.4%) were samples from the BT group. For the molds observed, 5/26 (19.2%) were from the BNT group and 3/14 (21.4%) were samples from the BT group. Of the BU negative samples (*N* = 11), growth was observed on 9 (81.8%) out of the 11 agar plates. Out of these, 7/9 (71.7%) were identified as yeast and 3/9 (33.3%) were mold. Thus 2/9 (22.2%) of the BU negative lesions had a mixed infection of both yeast and molds (Fig. 4).

Culture on M7H10 agar was maintained for 6 months as recommended [21]. Only 3 of the 51 decontaminated samples cultured on the M7H10 plates had visible characteristics of mycobacteria growth (rough and dry colonies). Further characterization of the colonies using microscopy revealed that 2 were non-acid fast club-shaped bacteria and the other was found to be acid fast coccoid shaped bacteria.

### Molecular identification of microbial isolates

**Fig. 5:**
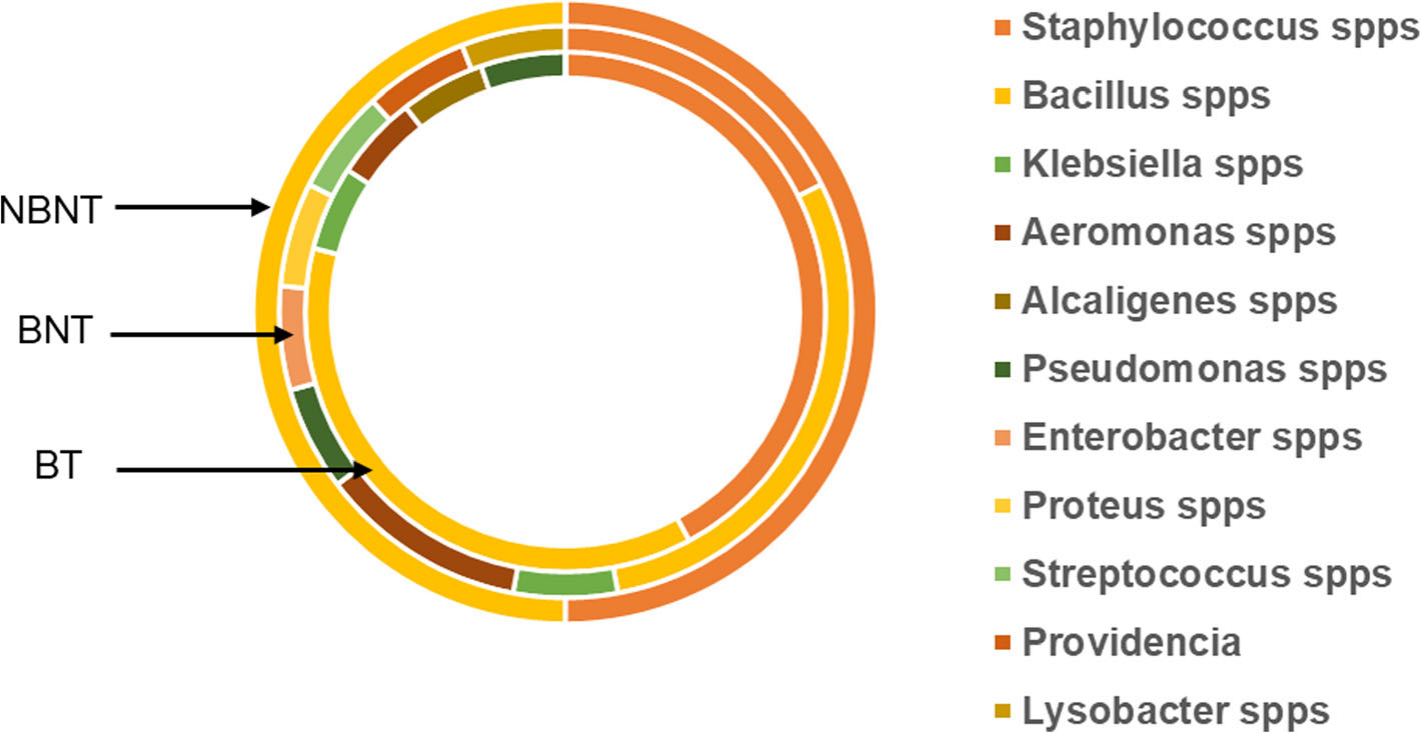
Bacteria species identified after amplicon sequencing. PCR amplicons for universal bacteria 16SrRNA were sequenced using the sanger method and queried using BLASTn for comparative sequence homology to identify bacteria present in the lesions. The outer circle represents bacteria identified from BU negative study group (NBNT), the middle circle represents BU patients that were not on treatment (BNT) and the inner circle represents the BU study group on the recommended antibiotic treatment (BT)

Further characterization of the DNA obtained from the isolated bacteria was achieved using PCR amplification of the universal bacterial 16SrRNA gene (Table 1), followed by amplicon sequencing to identify the microorganisms. Majority of the bacteria identified were *Staphylococcus* spp (24%), *Bacillus* spp (30%), *Pseudomonas* spp (6%) and *Alcaligenes* spp (6%). Other bacteria identified include *Proteus* spp, *Aeromonas* spp, *Enterobacter* spp, *Providencia* spp, *Klebsiella spp* among others. More bacteria diversity was observed among BU patients who were not yet on treatment (Fig. 5). PCR amplification followed by sequencing of the ITS region of the isolated fungi detected the presence of only 4 fungal species; *Penicillium citrinum*, *Trichodema longibrachiatum*, *Candida parapsilosis* and *Candida duobushaemulonii.*

**Table 1:** List of Primers used for PCR Amplification

Primers	Forward and reverse sequences (5’-3’)	Expected sizes (bp)	References
1_16SF	F-AGGAGGTGATCCAACCGCA R-AACTGGAGGAAGGTGGGAT	350	[22]
16SrRNA	MSHA-AAAAAGCGACAAACCTACGAG PA-AGAGTTTGATCCTGGCTCAG	620	[23]
IS*2404* (nested 1)	pGp1: AGGGCAGCGCGGTGATACGG pGp2: CAGTGGATTGGTGCCGATCGAG	400	[24, 25]
IS*2404* (nested 2)	pGp3: GGCGCAGATCAACTCGCGGT pGp4: CTGCGTGGTGCTTTACGCGC	200	[24, 25]
ITS	ITS1-TCCGTAGGTGAACCTGCGG ITS4-TCCTCCGCTTATTGATATGC	variable	[26]

PCR positive amplicons for the 16S hyper-viable region of mycobacteria were detected as *Corynebacterium* spp (80.4%); mainly *C. diphtheriae*, including others such as *C. aurimucosum*, *C. striatum*, *C. pollutisoli* and *C. jeikeium*, *Brevibacterium* spp. *JT-1* (6.6%), *M. marinum* (6.6%) and *M. ulcerans* (6.6%) after blasting on NCBI. Majority (60%) were identified on BU patients who had not begun the recommended antibiotic treatment with the rest distributed among BU patients under treatment (27%) as well as the non- BU groups (13%).

### Antimicrobial sensitivity profiles

**Fig. 6:**
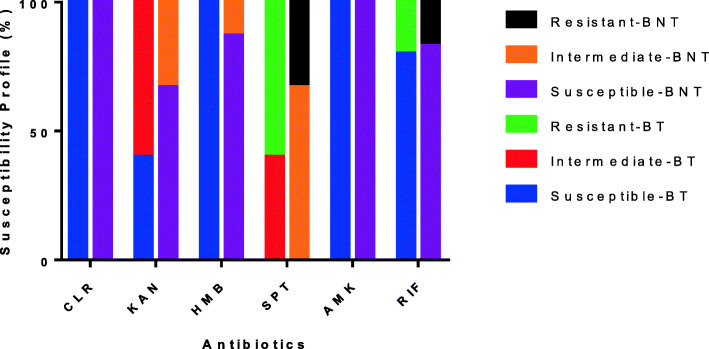
Efficacy of selected antibiotics against secondary bacteria that infect BU lesions. Clarithromycin (CLR), kanamycin (KAN), hygromycin B (HMB), streptomycin (STR), amikacin (AMK) and rifampicin (RIF) were tested against the recovered secondary bacterial isolates. The susceptibility profiles are shown for bacteria recovered from both patients who had commenced treatment (BT) and those who were not on treatment (BNT)

Clarithromycin (30 µg), kanamycin (30 µg), hygromycin B (30 µg), streptomycin (10 µg), amikacin (30 µg) and rifampicin (5 µg) are drugs known for treatment of BU and to suppress mycobacterial growth in *in-vitro* experiments. These drugs were tested against the isolated bacteria to determine their efficacy against the secondary bacteria recovered from the BU wounds. All the bacteria isolated in this study were susceptible to clarithromycin and amikacin (100%). This was followed by hygromycin B with 88.9% susceptibility and 11.1% intermediate. No bacterium was resistant to hygromycin B. Susceptibility to rifampicin which is recommended for BU treatment was similar to that of hygromycin B (82.2%) with resistance observed in 17.8% of the bacterial isolates. There were no intermediate zones observed. Kanamycin showed an average susceptibility of 51.1% and 48.9% intermediate zones. Streptomycin responded poorly as an antibiotic for treatment of secondary microbial infections. None of the bacteria were susceptible to streptomycin. However, intermediate zones were observed in 68.9% of the bacterial isolates and resistance in 17.8% of the bacteria isolates (Fig. 6). Resistance was mainly observed among the *Bacillus* spp, *Alcaligenes* spp and some *Staphylococci* spp. Generally, resistance to streptomycin and rifampicin was observed in both BNT and BT group. However, resistance was found to be higher among the BT group (60% for streptomycin, 20% for rifampicin) compared to the BNT group (33% for streptomycin, 17% for rifampicin). Nevertheless, bacteria isolated from the BT group were all susceptible to clarithromycin, hygromycin B and Amikacin whilst that for the BNT were susceptible to only clarithromycin and amikacin.

## Discussion

Characterization of microorganisms causing secondary infections in Buruli ulcer patients is necessary to understand their role in BU treatment efficacy and *M. ulcerans* pathogenesis. This information is also important in the holistic treatment and management of the disease. Although risk factors for bacteria colonization on BU lesions have not been extensively investigated, delayed treatment and insufficient wound assessment might contribute to the colonization and may prolong wound healing [27]. This study thus aimed at identifying secondary contaminating microorganism that may colonize BU wounds. This study also supported the assertion from other studies that other microbes, other than *M. ulcerans can* colonize BU wounds [11, 13, 28].

In comparison with PCR, we showed that acid-fast microscopy had a lower sensitivity in detecting MU (Fig. 2), which is consistent with similar observations in other studies [29]. This is partly due to the uneven distribution of *M. ulcerans* within the lesion and inability of the lesion swabbing technique to obtain a high enough bacterial load for visualization under a microscope. Additionally, majority of the patients enrolled in this study had already commenced antibiotic therapy and or wound dressing, therefore *M. ulcerans* loads and other secondary microorganisms could have been cleared from the lesions at the time of sampling. Moreover, some lesions with BU-like clinical presentation could have been misdiagnosed based on just the appearance of the lesion. Thus, detection of the insertion sequence 2404 which has a high sensitivity and specificity rate for BU case identification was used as cut-off for distinguishing BU verses non-BU cases.

The amplification of IS*2404* discriminated mycolactone producing mycobacteria (MPM) from nontuberculous mycobacteria (NTM), which is characterized by the detection of 16S rRNA hypervariable region. 16SrRNA positivity for mycobacteria was higher compared to IS*2404* positivity as expected [29, 30] and the 59 noncoding promoter region of the 16SrRNA gene for the identification and differentiation of mycobacterial species has been recommended [31]. It has been shown to offer several advantages over other target sequences such as the entire 16SrRNA region used in other studies. The 16SrRNA hypervariable region is also more polymorphic than the entire 16SrRNA coding region or portions of the *hsp65* gene that has been used in *Mycobacterium* spp detection, which resulted in an increased discriminatory power in this study. However, this region has also been found to detect other Actinobacteria such as *Corynebacteria* spp as observed in this and other similar studies [24].

IS*2404* is now considered to be non-specific for *M. ulcerans* due to the detection of this insertion sequence in a number of other mycobacterial species such as *M. liflandii* and *M. marinum* DL [32]. This emphasizes the need for sequencing these targets for accurate species identification. Results from this study suggests the combination of molecular and culturing methods to provide a better characterization of the microbial diversity of chronic wounds. These will help expand our understanding of how microbiology impacts chronic wound pathology and healing.

Several studies have described bacteria that infect lesions as mostly normal skin flora and are therefore, non-pathogenic [33]. Majority of the *Staphylococci* identified in this study were mannitol fermenters which are considered pathogenic. *S. aureus*, abundantly found in BU lesions of this study has been found to be the predominant bacteria isolated from several BU wounds [14, 27, 28]. *S. aureus* have been found to harbor a diverse repertoire of virulence factors such as α-hemolysin, and other mobile genetic elements (MGEs) including genomic island prophages and pathogenicity islands [34]. These factors are known promote immune evasion, and development of superantigens which may enhance the persistence and survival of the bacteria in the lesion [34]. Therefore pathogenic *Staphylococci* may worsen the condition of the ulcers and may delay the healing process of the lesion [35–37].

Gram negative bacteria have been found to be a major contributor of secondary infections in BU lesions [28]. Both gram negative lactose and non-lactose fermenters were isolated from the lesions. Even though *E. coli* is a common microbial infection in BU endemic areas, we did not identify any in the microbial isolates sequenced, nevertheless, *Pseudomonas aeruginosa* was identified. Lactose negative bacteria which includes *P. aeruginosa* usually cause secondary bacterial infection especially in difficult-to-heal wounds such as skin ulcers [30]. *P. aeruginosa* have been identified to dominate in both pre-treatment and post-treatment of BU [10] and is often cited as a source of delay in wound healing [38, 39]. *Klebsiella* spp are well known normal flora of the human mouth and the gastrointestinal tract and are scarcely found on the skin[40]. *Klebsiella* spp and *Proteus* spp have also been associated with wound infections [41].

*Staphylococcus aureus* and *Alcaligenes* spp are usually identified as secondary infections in wounds. Both bacteria have been identified as common nosocomial secondary infections among many of the patients with the disease [42]. In a recent study conducted in Ghana, *Staphylococcus* spp, which included methicillin resistant *S. aureus* were identified as nosocomial secondary infections among BU patients with possible transmission from healthcare workers, hospital equipment for wound dressing such as the forceps [13, 43]. *Alcaligenes* spp have also been recognized as opportunistic emerging infectious gram-negative bacterial species that can affect immunosuppressed patients [44].

*Bacillus* spp belonging to the *B*. *cereus* group of bacteria were also identified after sequencing. Bacteria belonging to this group are facultatively anaerobic and spore-forming bacteria. They are known to be frequently distributed in a wide range of environmental niche [45]. Strains from the *B. cereus* group are commonly found as part of the plant and soil microbiome [46]. *B. cereus* infections of human and domestic animals have also received recognition and increasing number of infections from wounds [47] and insect bites [48] have been reported to have been colonized by these bacteria. It has been noticed mainly in immunosuppressed tissues of wounds [49]. It is therefore, not surprising to be noted as a secondary microorganism in BU wounds.

Due to the similarities between *Corynebacterium* and *Mycobacterium* spp, *Corynebacterium* is sometimes used in the study of *Mycobacteria* [24, 50]. This could explain why many *Corynebacterium* spp were noticed after sequencing of the 59 noncoding promoter region of the 16SrRNA gene that targets mycobacteria. These bacteria have been occasionally sequenced using the 16SrRNA primer for *Mycobacteria* [51]. However, unlike *Mycobacteria*, *Corynebacterium* spp are non-acid fast bacteria that can grow on Middlebrook7H9 medium. They are soil dwelling microorganisms that are usually infectious and pathogenic. Some like *C. ulcerans* can manifest infections on the skin that closely resembles that of *M. ulcerans* [52–54]. Slow healing wounds have been confirmed to be usually colonized by *Corynebacterium* spp [55]. This could explain why a number of *Corynebacterium* spp were identified from the microbial isolates.

Majority of the bacteria identified were pathogenic and opportunistic bacteria that live in muddy areas and in dirty water bodies. Almost all the bacteria are soil dwellers that easily have access to the BU lesion during activities like playing, farming, or mining. Very few were normal body flora that may delay healing of BU lesions.

Fungal elements have been isolated from several ulcers. The most common species identified are *Candida, Cryptococcus, Trichosporon* and *Rhodotorula* spp [56]. Thus, fungi identified in this study through staining and culturing was no surprise. *Candida duobushaemulonii* is part of the *Candida haemulonii* complex (*C. haemulonii, C. haemulonii var. vulnera* and *C. duobushaemulonii*). This group of Candida are noted for their high antifungal resistance, including high minimum inhibitory concentrations (MICs) of amphotericin B and cross-resistance to azole compounds [57, 58]. Their presence in the BU lesions may also delay healing. *Candida parapsilosis* has been described as an opportunistic pathogen. *Trichoderma* species are common fungal species usually found in humid soil, decaying wood, and water-related sites. *T. longibrachiatum*, have been identified as causative agents of infections in immunosuppressed hosts [59]. The BU patients may have been infected with the fungi through a variety of sources which may include the farm (a high percentage of the BU study group were farmers), homes and even at the place of treatment such as the hospitals and community treatment centers [13] (Fig. 1). Mycolactone has also been found to enhance spore germination and chemoattractant effects on some fungal species [15]. This may explain the reason for fungal detection in BU lesions as well as the major contaminant for MU culture.

WHO recommends a combination drug of rifampicin and streptomycin or clarithromycin for 8 weeks for the treatment of BU [60]. Although these have been generally effective in treatment, drugs for secondary microbial infections of BU patients have not yet been recommended. Resistance to streptomycin and rifampicin have been reported for *Bacillus* spp and the *Staphylococcus* spp. The *Bacillus* spp mostly form spores in harsh conditions and this may be the reason for their resistance to the drugs [61]. Streptomycin used for the treatment of secondary BU infection may be unsuitable due to the high level of resistance of secondary bacteria as observed in a similar study [14]. *M. ulcerans* is sensitive not just to streptomycin and rifampicin but also to clarithromycin [62–64].

Since all the secondary bacteria identified in this study were susceptible to clarithromycin and amikacin, their continuous use in chemotherapy could serve as treatment for the secondary bacterial infections of BU. The bactericidal activity of the combination rifampicin-clarithromycin against *M. ulcerans* has been found to be similar to that of rifampicin-streptomycin [10]. This may likely be a better option for treatment for BU since the secondary bacteria were all susceptible to clarithromycin. To corroborate this observation recent clinical trial in Ghana found rifampicin-clarithromycin combination as a suitable treatment for BU treatment [63, 64].

Unfortunately, the long distances to the nearest health center deter most BU patients from seeking treatment. A higher susceptibility was observed among the microorganisms isolated from Buruli ulcer patients who had already began antibiotic treatment (BT) compared to those who had not (BNT). It was also noticed that resistance to the recommended antibiotics, (rifampicin and streptomycin) was higher among the BT compared to the BNT group. High bacterial loads have been found to increase significantly even after treatment of the disease [13]. This could explain the higher level of resistance identified among the BU group who had already began antibiotic treatment. However, other studies, suggest that BU patients with small lesions (category 1) are more likely to heal quicker compared to others if treatment commences early enough [63–65].

The type of secondary microbial infection on a BU patient may not necessarily depend on factors such as geographical area, personal hygiene, type of treatment, among others. In recent studies majority of the secondary bacterial infection in BU patients were like those found in this study [10, 27, 28]. Sequence analysis revealed that majority of the bacteria identified on the BU lesions are Gram positive and negative rods compared to the cocci. Further studies are needed to compare secondary bacterial infection from the surface of BU lesions and from the undermined edges where *M. ulcerans* are predominantly found.

## Conclusions

Secondary microbial infections among BU patients are common; with most being of bacterial etiology. The common bacteria identified are *Staphylococcus* spp, *Bacillus* spp, *Alcaligenes* spp *and Pseudomonas* spp. Predominant bacteria present among both BU and Non-BU patients were found to be *Staphylococcus* spp and *Bacillus* spp. Fungal species identified were mainly *Candida* spp. The selected bacteria were all susceptible to amikacin and clarithromycin, however, high resistance was observed with streptomycin. These microbial infections may delay healing and increase resistance to antibiotics administered to BU patients.

The attitude of patients to BU may also lead to secondary microbial infections on the lesions. Most patients report very late for treatment. Some even worsen the lesion using non-aseptic herbal medications, which are additional source of secondary microbial infections. Although risk factors for bacterial wound colonization have not been thoroughly studied to date, delayed treatment and insufficient wound management might contribute to colonization and prolonged wound healing. There is thus, the need to seek medical help as soon as possible when signs of BU are noticed. Home dressing and traditional healing should be discouraged if possible. Since many of the bacteria identified are soil dwellers, BU patients should be educated on the importance of personal hygiene and the wearing of protective clothing when attending to their various occupations to prevent the proliferation of these secondary microorganisms.

## Methods

### Study design

This study was cross-sectional and designed to identify secondary microorganism from suspected BU lesions within a period of 2 years, from June 2014 to June 2016.

### Study communities

An active case search for suspected and unreported BU cases was conducted in all the study communities in the Amansie Central District of the Ashanti Region, Ghana. Suspected BU cases [15] who reported to the St. Peter’s hospital and had had their cases confirmed with acid fast staining but not IS*2404* (the gold standard for BU confirmation) were included in this study. In all, a total of fifty-one [55] suspected BU cases were identified in the district. The 51 participants all consented to the study and were recruited from 25 BU endemic communities, with majority from Jacobu (56%), Krofrom (20%), Homase (16 mm %) and Donkoase (12%). A questionnaire was administered to each participant to gather basic demographic information including age, residence, occupation, treatment status and category of lesion. Majority of the inhabitants in the Amansie Central district are farmers, with some inhabitants involved in small-scale surface gold mining (galamsey) along the rivers which run through the district. At least one functioning borehole was found in each community. Nevertheless, the inhabitants still fetched water from the river for domestic and agricultural activities [24].

### Sample collection

Multiple swabs [3] were taken from all suspected BU cases having clinical presentation of BU (from the undermined edges of open lesions and from the surface of the lesion) and were placed in labeled sterile 15 mL sterile falcon tubes. The samples were preserved with ice packs in a cooler and transported to the St. Peters Hospital (Jacobu) for temporal storage at 4ºC and onward transport in cold storage to the laboratory at the Department of Biochemistry, Cell and Molecular Biology in Accra.

### Sample processing for laboratory analyses

The swabs were processed for microscopy, culture and DNA extraction. Briefly, 2 mL of 1X Phosphate Buffer Saline (PBS) was added to the 15 mL falcon tube containing the swab and the tube was vortexed for 5 minutes to dislodge microbial cells from the swabs. The mixture was then used for acid-fast staining (10 µL), culturing (10 µL) and DNA extraction (200 µL). The remaining mixture was preserved at 4ºC for later use.

### BU case confirmation

To detect *M. ulcerans* infections, acid-fast staining was performed on samples stored in PBS, following the protocol from BD Biosciences. This was then followed by PCR confirmation. Briefly, genomic DNA was extracted from 200 µL of the PBS mixture using the Zymogene Quick-DNA ^TM^ Miniprep Kit. The purified genomic DNA was then used for PCR targeting the IS*2404* sequence. This insertion sequence is the WHO recommended molecular diagnostic marker for BU case confirmation and is present in over 200 copies on the genome of mycolactone producing mycobacteria [20]. Patients with confirmed *M. ulcerans* infection, i.e. BU, were referred to their respective Community Health Centers for treatment.

### Culture isolation of secondary microorganisms

To isolate secondary microorganisms from the lesions, the samples stored in PBS were cultured on each of the following media : Luria Bertani (LB) agar at 37^o^C (for the isolation of general bacteria), MacConkey agar at 37^o^C (for the isolation of Gram negative and enteric bacteria), Mannitol salt agar at 37^o^C (for the isolation of *Staphylococci* and *micrococci* bacteria), Sabouraud dextrose agar supplemented with chloramphenicol at 30^o^C (for the isolation of fungal elements) and Middlebrook 7H10 agar at 32^o^C (for the isolation of *Mycobacterium* spp). All bacteria were cultured for 16 to 24 hours, 5 days to 1 week for fungal growth and up to 6 months for mycobacteria. All bacterial growth observed were either Gram or acid-fast stained to confirm type of bacteria. All fungal species were also observed under a microscope using the wet mount technique.

### Molecular identification of microorganisms

To identify the cultured bacterial isolates, we amplified and sequenced the 16SrRNA using previously published primers including the universal bacteria [22], and *Mycobacterium*-specific 16SrRNA primers [23]. To distinguish the mycolactone producing mycobacteria (MPM) from other mycobacteria we amplified and sequenced the IS*2404* gene as described previously [24, 25]. To identify infecting fungi, we amplified and sequenced the Internal transcribed Spacer region (ITS) as described elsewhere [26]. All primers used are described in Table 1. All PCR amplicons were Sanger-sequenced (Macrogen Inc, Netherlands) and quality filtering was done by trimming the ends of the nucleotide sequence using Finch TV chromatogram viewer (version 1.4.0). The sequence reads were queried using the BLASTn program on NCBI (https://blast.ncbi.nlm.nih.gov/Blast.cgi). The E-value for homology comparison among the species was chosen to be below 10^− 4^ or 0.0001. Search queries with first hits > 90% nucleotide similarity was used to characterize isolates [66].

### Antibiotic susceptibility testing

Following the molecular identification of the bacterial isolates, we performed drug sensitivity testing to assess their susceptibility to the commonly used anti-mycobacterial drug on Mueller Hinton agar plate and incubated at 37^o^C for 18 hours. The antibiotic used were amikacin (30 µg), kanamycin (30 µg), clarithromycin (30 µg), hygromycin B (30 µg), streptomycin (10 µg) and rifampicin (5 µg). These tests were performed using the Kirby Bauer protocol [67].

## Data Availability

All data generated or analyzed during this study are included in this published article and its supplementary information files.

## Abbreviations

BU: Buruli ulcer
MPM: Mycolactone Producing Mycobacteria
PBS: Phosphate Buffer Saline
NCBI: National Center for Biotechnology Information
IS*2404*: Insertion Sequence 2404
*spp*: Species
CLR: Clarithromycin
HMB: Hygromycin
AMK: Amikacin
KAN: Kanamycin
SPT: Streptomycin
RIF: Rifampicin
BNT: Buruli ulcer cases not under treatment
BT: Buruli ulcer cases under treatment
